# Hsa_circ_0039569 facilitates the progression of endometrial carcinoma by targeting the miR-197/high mobility group protein A1 axis

**DOI:** 10.1080/21655979.2022.2027060

**Published:** 2022-02-07

**Authors:** Yi Zhou, Anyi Pan, Yudong Zhang, Xinchun Li

**Affiliations:** a Third Department of Gynecology and Oncology, Hunan Cancer Hospital, Changsha, Hunan Province, China; b Department of General Medicine, Second Xiangya Hospital, Central South University, Changsha, Hunan Province, China

**Keywords:** Endometrial carcinoma, HMGA1, hsa_circ_0039569, miR-197

## Abstract

Circular RNAs are novel regulators in endometrial carcinoma. Hsa_circ_0039569 was reportedly upregulated in endometrial carcinoma; however, the functional roles and mechanisms of hsa_circ_0039569 need further investigation. Therefore, we used quantitative real-time PCR (qRT–PCR) to determine the mRNA levels of hsa_circ_0039569, miR-197 and high mobility group protein A1 (HMGA1). The protein level of HMGA1 was determined by Western blot. Cell Counting Kit-8 and colony formation assays were used to assess cell proliferation. Cell migration was measured via wound healing and Transwell assays. Transwell assay was also performed to determine cell invasion ability. Direct binding of the indicated molecules were verified by RNA binding protein immunoprecipitation (RIP) assay and dual luciferase reporter assay. The results revealed that hsa_circ_0039569 and HMGA1 were elevated, while miR-197 was downregulated in endometrial carcinoma. Moreover, hsa_circ_0039569 was positively correlated with the expression of HMGA1 and was negatively correlated with the level of miR-197. In addition, hsa_circ_0039569 facilitated the proliferation, migration and invasion of endometrial carcinoma cells. The underlying mechanism is that hsa_circ_0039569 serves as a sponge of miR-197 to repress the inhibitory effect of miR-197 on HMGA1. Furthermore, the miR-197/HMGA1 axis was implicated in endometrial carcinoma progression accelerated by hsa_circ_0039569. Collectively, hsa_circ_0039569 may promote the development of endometrial carcinoma by serving as an endogenous sponge of miR-197, increasing HMGA1 expression and identifying a novel target for endometrial carcinoma treatment.

## Introduction

Endometrial carcinoma is a common gynecological malignancy in women worldwide [1]. Risk factors, such as postmenopausal conditions, long-term use of estrogen and high blood pressure, play critical roles in the pathogenesis of endometrial carcinoma [2]. Although considerable progress has been made in the understanding and treatment of endometrial carcinoma, there are currently no effective biomarkers [3]. Therefore, identifying the detailed mechanisms and finding reliable molecular targets to accelerate the development of diagnosis and treatment in endometrial carcinoma is urgently needed.

Circular RNA (circRNA) is a type of new noncoding RNA with a covalently closed structure [4]. CircRNAs play a critical role in the progression of many types of cancers, including endometrial carcinoma [5]. Liu et al. found that circTNFRSF21 facilitated the pathogenesis of endometrial carcinoma by targeting miR-1227 [6]. Has_circ_0061140 was also show to enhance the progression of endometrial carcinoma by targeting the miR-149/signal transducer and activator of transcription 3 (STAT3) axis [7]. In addition, a study by Wei et al. revealed that circ_0000043 mediated the malignant phenotypes of endometrial carcinoma by regulating the miR-1271-5p/catenin delta 1 (CTNND1) axis [8]. Moreover, hsa_circ_0005797 was also shown to be a regulator in endometrial carcinoma [9]. A recent study found that hsa_circ_0039569 was significantly upregulated in grade 3 endometrial carcinoma [10]. However, the functional roles and underlying mechanisms of hsa_circ_0039569 need further investigation.

MiR-197 is a microRNA (miRNA) that is dysregulated in several types of cancers. In prostate cancer, miR-197 was downregulated, and overexpression of miR-197 suppressed cell proliferation via voltage-dependent anion channel 1 (VDAC1)/AKT/β-catenin signaling [11]. In ovarian cancer cells, miR-197 attenuated the progression of epithelial mesenchymal transition by inhibitingATP binding cassette subfamily A member 7 (ABCA7) [12]. Inconsistently, a study revealed that miR-197 facilitated invasion of tumor cells by regulating insulin-like growth factor-binding protein 3 (IGFBP3) in colorectal cancer [13]. Additionally, miR-197 was sponged by hsa_circ_0002577 in endometrial carcinoma and involved in the activation of Wnt/β-catenin signaling [14]. MiR-197 was also shown to be sponged by circ_ZNF609 in cervical cancer [15]. However, a more detailed mechanism of miR-197 in endometrial carcinoma should be investigated. Our preliminary study revealed a potential binding site of hsa_circ_0039569 with miR-197, suggesting that miR-197 might mediate the function of hsa_circ_0039569 in endometrial carcinoma.

In the present study, we hypothesized that hsa_circ_0039569 might serve as a sponge of miR-197 to facilitate the progression of endometrial carcinoma. The aim and goal of this study was to illustrate the oncogenic role and mechanism of hsa_circ_0039569 in endometrial carcinoma. Our data revealed that hsa_circ_0039569 was upregulated in endometrial carcinoma and that inhibition of hsa_circ_0039569 impaired the progression of endometrial carcinoma *in vitro*. We demonstrated a novel miR-197/HMGA1 axis modulated by hsa_circ_0039569. Collectively, our findings might provide new insight into endometrial carcinoma treatment.

## Materials and methods

### Clinical tissue samples

Endometrial carcinoma tissues and matched adjacent normal tissues were collected from 36 patients with a hysterectomy at Hunan Cancer Hospital from March 2018 to May 2020 as previously described [6]. These patients did not receive chemotherapy or radiotherapy before the operation. The study was approved by the Ethics Committee of Hunan Cancer Hospital. Table 1 shows the patients’ clinical characteristics.

**Table 1.** The characteristics of the endometrial carcinoma patients

Parameters	Group	Cases (n)
Age (years)	≥50	23
	<50	13
Lymphatic metastasis	Positive	20
	Negative	16
FIGO stage	I–II	15
	III–IV	21
Pathological type	Endometrioid	36
	Non-endometrioid	6
Histological grade	G1	18
	G2+ G3	18
Menstruation	Non-menopause	13
	Menopause	23
Estrogen receptor	Positive	11
	Negative	25
Progesterone receptor	Positive	14
	Negative	22

### Cell culture

Endometrial carcinoma cell lines, including HEC-1-B, AN3-CA, KLE, HEC1-A, Ishikawa, and hEEC (human endometrial endothelial cells), were purchased from ATCC or Shanghai Cell Bank of the Chinese Academy (Shanghai, China). Cells were cultured in RPMI-1640 (Invitrogen, USA) with 10% FBS (Gibco, USA) in 5% CO_2_ at 37°C as previously described [6].

### Cell transfection

Small interfering RNA (siRNA) targeting hsa_circ_0039569 (si-circ, 1: TCCTTTAAAATAGTGCCCCTA; 2: ATCCTTTAAAATAGTGCCCCT) and HMGA1 (si-HMGA1: ACTGGAGAAGGAGGAAGAG), miR-197 inhibitor (GCUGGGUGGAGAAGGUGGUGAA), and their negative controls were synthesized by Guangzhou RiboBio (China). The overexpression vectors pcDNA-hsa_circ_0039569 (ov-circ) and pcDNA-HMGA1 (ov-HMGA1) were constructed by Shanghai GenePharma. HEC-1-B or Ishikawa cells were transfected with the above plasmid via Lipofectamine 3000 (Invitrogen) as previously reported [16]. Transfection efficiency was detected by qRT–PCR or Western blot.

### Cell proliferation and apoptosis measurements

Colony formation assays and CCK-8 tests were performed to assess cell proliferation according to a previous report [17]. In the colony formation assay, transfected cells were resuspended in RPMI-1640 and seeded into 6-well plates at a density of 700 cells/mL for 2 weeks. Once colonies formed, cells were fixed using methanol and stained with crystal violet for subsequent number counting. For the CCK-8 assay, cells (2 × 10^3^/well) were cultured in 96-well plates. After transfection, 10 μL CCK8 reagent (Beyotime, China) was added to fresh medium and incubated with treated cells for 2 h. Then, absorbance was measured at 450 nm. For apoptosis detection, cells were obtained and stained with 5 μL PI and FITC-Annexin V (Thermo Fisher Scientific) for 15 min, and FACS was used to assess the apoptosis rate.

### Quantitative real-time PCR (qRT–PCR)

qRT–PCR was performed as previously described [17]. Total RNA was extracted by TRIzol (Invitrogen) and reverse transcribed into cDNA using a PrimeScript RT reagent kit (TaKaRa, Japan). A TaKaRa PCR kit was used for qRT–PCR under the following conditions: 95°C for 10 s and 60°C for 60 s, repeating for 40 cycles. The RNA expression level was calculated using the 2^−ΔΔCt^ method normalized to U6 or GAPDH. A Cytoplasmic & Nuclear RNA Purification Kit (Norgen Biotek, Canada) was used for nucleo-cytoplasmic separation according to the manufacturer’s instructions. The primer sequences used in this study were as follows: hsa_circ_0039569 forward: 5’-AAAATAGTGCCCCTACGGCG-3’, hsa_circ_0039569 reverse: 5’-GGCAGACGGTAACGGACGTA-3’; miR-197 forward: 5’-GCCTTCACCACCTTCTCCA-3’, miR-197 reverse: 5’-CGGCCCAGTGTTCAGACTAC-3’; HMGA1 forward: 5’-CCTCCAAGCAGGAAAAGGAC-3’, HMGA1 reverse: 5’-CTTCCTGGAGTTGTGGTGGT-3’; U6 forward: 5’-CTCGCTTCGGCAGCACA-3’, U6 reverse: 5’-AACGCTTCACGAATTTGCGT-3’; and GAPDH forward: 5’-AGGTCGGAGTCAACGGATTT-3’, GAPDH reverse: 5’-TGACGGTGCCATGGAATTTG-3’.

### Western blotting

Western blotting was performed as previously described [17]. Tissue or total cell proteins were extracted by PIPA reagent (Yeasen, China), and proteins were separated by 10% SDS–PAGE and then transferred to PVDF membranes. HMGA1 primary antibody (Cell Signaling Technology, #7777) was used and incubated with PVDF membranes overnight at 4°C. Next, the membranes were incubated with anti-rabbit secondary antibody (Cell Signaling Technology, #7074) for 1 h. Protein-specific bands were visualized using the BioSpectrum Imaging System, and the expression level was analyzed via ImageJ software.

### Transwell assay

Transwell assays were performed as previously described [18]. Cells were resuspended in medium without FBS at a density of 10^5^ cells/mL. Then, 700 mL medium with 10% FBS was added to the lower chambers, and 500 μL cell suspension was seeded in the upper Transwell chambers (Corning, 8 μm pore). For the invasion assay, Matrigel (Corning) was used to coat the Transwell chambers for 3 h before cells were seeded. After 48 h, in the upper chamber, cells were swabbed, and cells adhered to the lower chamber were fixed with 4% formaldehyde and stained with crystal violet. Finally, chambers were visualized under a microscope for cell counting.

### Wound healing assay

A wound healing assay was performed as previously described [19]. After HEC-1-B or Ishikawa cells were transfected for 48 h, wounds were formed using a 200 μL tip, and dishes were washed with PBS. The width of wounds was measured at 0 h and 24 h, and the relative migration rate was calculated.

RNA immunoprecipitation (RIP) assay

RIP was performed as previously described [7]. Cell lysates of HEC-1-B or Ishikawa cells were prepared using RIP lysis buffer (Merck, UK) and incubated with specific Ago2 (Abcam, UK) antibody or IgG antibody (Abcam). The antibodies were coupled with Sepharose beads. The samples were exposed to a magnet, and the protein complex was collected using TRIzol reagent for qRT–PCR determination.

### Dual-luciferase reporter assay

A dual-luciferase reporter assay was performed as previously described [7]. Circular RNA Interactome (https://circinteractome.nia.nih.gov/) and starBase (http://starbase.sysu.edu.cn/) analyses were performed to predict the potential binding sites for miR-197 in hsa_circ_0039569 and HMGA1, respectively. The sequences were cloned into the pGL3 vector (GeneChem, China) to construct pGL3-WT and pGL3-MUT for HMGA1 or hsa_circ_0039569. After cotransfection of the reporter plasmid and NC/miR-197 inhibitor or miR-NC/miR-197 mimics, the luciferase activity was examined using a Luciferase Reporter Gene Assay kit (Yeasen).

### Statistical analysis

Data for statistical analysis were obtained from three independent experiments analyzed using GraphPad Prism 8.0 (La Jolla, CA, USA) and are presented as the mean ± SD. Student’s *t* tests and one-way ANOVA were applied for significant differences. The correlation in the expression levels of hsa_circ_0039569, miR-197 and HMGA1 was reflected via Pearson’s correlation coefficient test. *P* < 0.05 was accepted as statistically significant.

## Results

**Figure 1.**
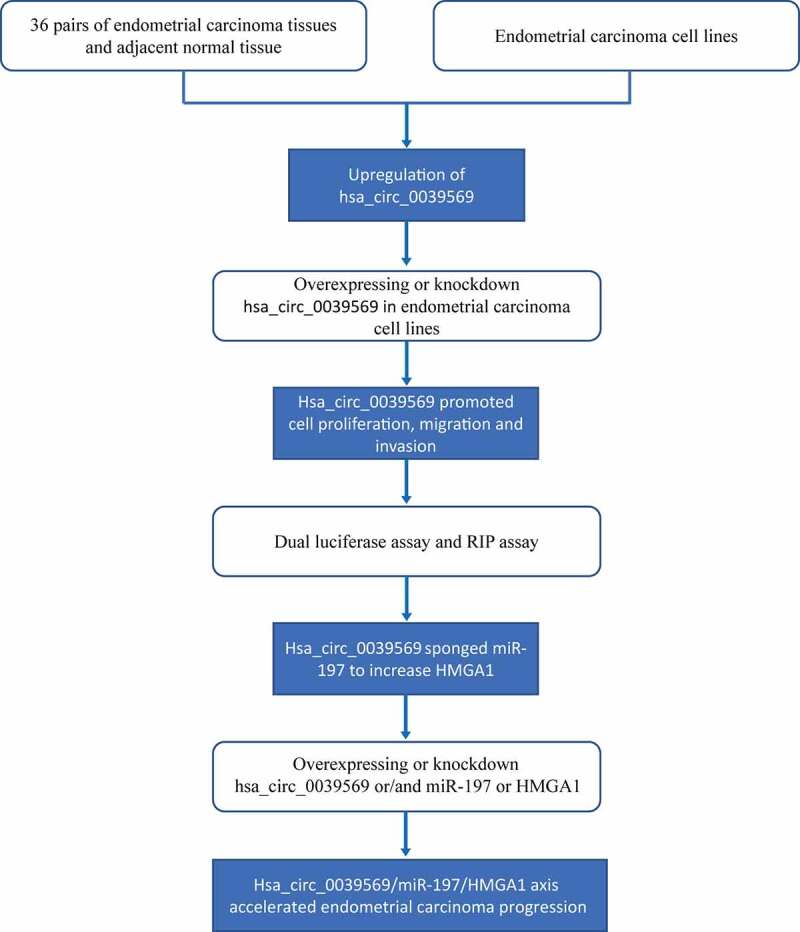
A flow chart diagram of the design for this study.

The present study illustrates the oncogenic role and mechanism of hsa_circ_0039569 in endometrial carcinoma, and the flow chart diagram of the design is illustrated in Figure 1. We found that hsa_circ_0039569 promoted cell proliferation, migration and invasion in endometrial carcinoma, which occurred at least partially through sponging miR-197 to decrease the inhibitory effect of miR-197 on HMGA1.

### Hsa_circ_0039569 was elevated in endometrial carcinoma

**Figure 2.**
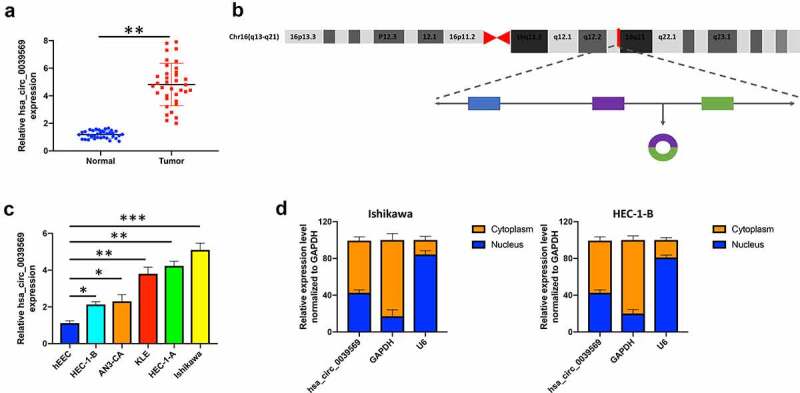
**Hsa_circ_0039569 was elevated in endometrial carcinoma**. (a) Expression of hsa_circ_0039569 in normal tissues and endometrial carcinoma tissues was detected by qRT–PCR. (b) Illustration of the generation of hsa_circ_0039569 from the CCL22 gene. (c) Expression of hsa_circ_0039569 in endometrial carcinoma cell lines was detected by qRT–PCR. (d) The distribution of hsa_circ_0039569 in the nucleus and cytoplasm was detected by qRT–PCR. * denoted *P* < 0.05, ** denoted *P* < 0.01, *** denoted *P* < 0.001.

We first investigated the expression of hsa_circ_0039569 in endometrial carcinoma. As shown in Figure 2a, hsa_circ_0039569 was notably upregulated in endometrial carcinoma tissues. The formation of hsa_circ_0039569 is illustrated in Figure 2b. To confirm the result in clinical samples, we also determined the level of hsa_circ_0039569 in endometrial carcinoma cell lines. The results indicated that, compared to the human endometrial epithelial cell line, hsa_circ_0039569 was increased in endometrial carcinoma cell lines. Among tumor cell lines, Ishikawa showed the highest hsa_circ_0039569 expression, while HEC-1-B showed the lowest (Figure 2c). Therefore, these two cell lines were used for subsequent experiments. Moreover, we found that hsa_circ_0039569 was abundantly located in the cytoplasm of HEC-1-B and Ishikawa cells (Figure 2d). Collectively, hsa_circ_0039569 was shown to be elevated in endometrial carcinoma.

### Hsa_circ_0039569 regulated cell proliferation in endometrial carcinoma

**Figure 3.**
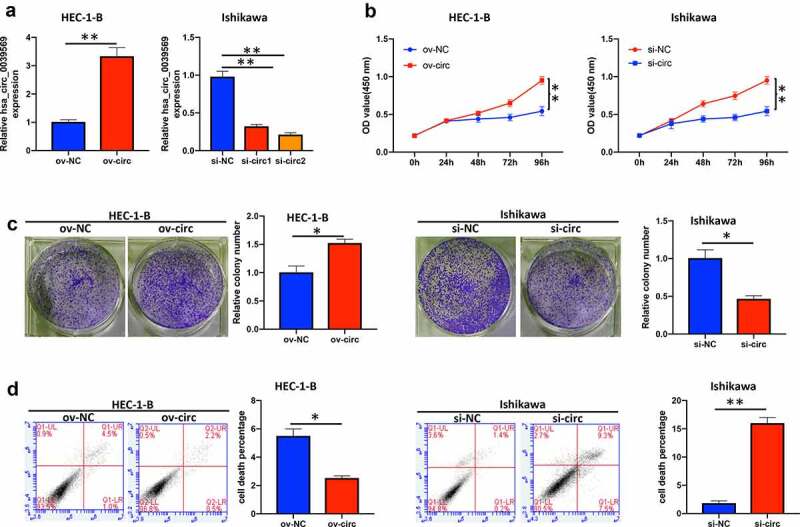
**Hsa_circ_0039569 regulated cell proliferation in endometrial carcinoma**. Hsa_circ_0039569 was overexpressed in HEC-1-B cells and was knocked down in Ishikawa cells. (a) Expression of hsa_circ_0039569 in HEC-1-B and Ishikawa cells was detected by qRT–PCR. (b) Cell viability was determined by CCK8. (c) A colony formation assay was performed to determine cell proliferation. (d) Cell apoptosis was evaluated by flow cytometry. * denoted *P* < 0.05, ** denoted *P* < 0.01.

To investigate the functional roles of hsa_circ_0039569 in endometrial carcinoma cells, we overexpressed hsa_circ_0039569 in HEC-1-B cells and knocked down hsa_circ_0039569 in Ishikawa cells. The efficiency of transfection was validated by qRT–PCR. Overexpression of hsa_circ_0030569 (ov-circ) increased its level, and si-circ2 (siRNA targeting hsa_circ_0039569) showed a better knockdown effect in Ishikawa cells and was applied to the following experiments (Figure 3a). As shown in Figure 3b, overexpression of hsa_circ_0039569 enhanced the viability of HEC-1-B cells, while hsa_circ_0039569 knockdown impaired that of Ishikawa cells. Moreover, the colony formation assay showed consistent results, indicating that hsa_circ_0039569 promoted the proliferation of endometrial carcinoma cells (Figure 3c). Additionally, we also found that hsa_circ_0039569 upregulation reduced the apoptosis rate of HEC-1-B cells, whereas hsa_circ_0039569 repression led to a substantial increase in apoptosis in Ishikawa cells (Figure 3d). Taken together, these results suggested that hsa_circ_0039569 positively regulated the proliferation of endometrial carcinoma cells.

### Hsa_circ_0039569 mediated the migration and invasion of endometrial carcinoma cells

**Figure 4.**
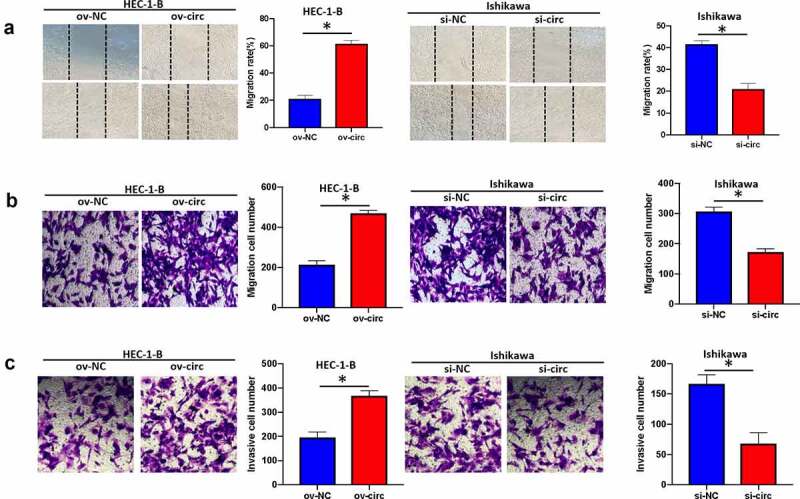
**Hsa_circ_0039569 mediated the migration and invasion of endometrial carcinoma cells**. Hsa_circ_0039569 was overexpressed in HEC-1-B cells and knocked down in Ishikawa cells. (a and b) Cell migratory ability was assessed by wound healing (a) and Transwell (b) assays. (c) Cell invasive ability was assessed by Transwell assay. * denoted *P* < 0.05.

We next investigated whether hsa_circ_0039569 mediated the migration and invasion of endometrial carcinoma cells. As indicated by wound healing assay and Transwell assay, overexpression of hsa_circ_0039569 increased the migrative ability of HEC-1-B cells, while hsa_circ_0039569 suppression showed the opposite effect in Ishikawa cells (Figures 4a and 4b). Additionally, we also validated that hsa_circ_0039569 facilitated the invasion of endometrial carcinoma cells. As shown in Figure 4c, hsa_circ_0039569 overexpression enhanced the invasion of HEC-1-B cells, and hsa_circ_0039569 knockdown impaired the invasion of Ishikawa cells. These findings demonstrated that hsa_circ_0039569 facilitated cell migration and invasion in endometrial carcinoma.

### Hsa_circ_0039569 served as a sponge of miR-197

**Figure 5.**
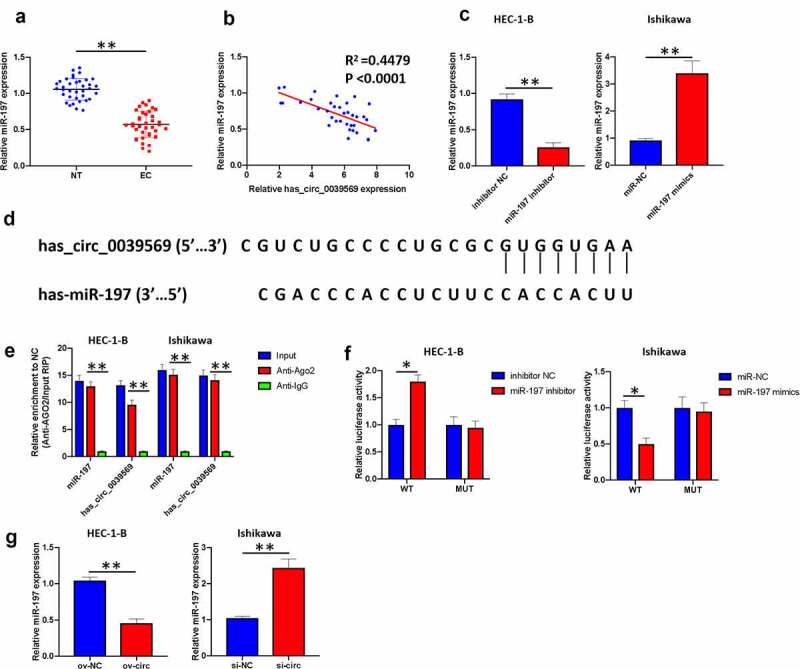
**Hsa_circ_0039569 acts as a sponge of miR-197**. (a) Expression of miR-197 in normal tissues and endometrial carcinoma tissues was detected by qRT–PCR. (b) The correlation between miR-197 and hsa_circ_0039569 was assessed by Pearson’s correlation coefficient test. (c) MiR-197 was silenced in HEC-1-B cells and overexpressed in Ishikawa cells. (d) Illustration of the predictive binding site between miR-197 and hsa_circ_0039569. (e) RIP assay was performed to validate the direct binding of miR-197 and hsa_circ_0039569 on Ago2. (f) A dual luciferase reporter assay was performed to verify the interaction of miR-197 and hsa_circ_0039569. (g) Hsa_circ_0039569 was overexpressed in HEC-1-B cells and knocked down in Ishikawa cells. Expression of miR-197 was detected by qRT–PCR. * denoted *P* < 0.05, ** denoted *P* < 0.01.

Subsequently, we explored the potential mechanism of hsa_circ_0039569 in modulating endometrial carcinoma. MiR-197 is downregulated in endometrial carcinoma [14]. Consistently, we found that miR-197 was decreased in the enrolled endometrial carcinoma samples (Figure 5a). Moreover, we found that the expression level of hsa_circ_0039569 in endometrial carcinoma was negatively correlated with miR-197 (Figure 5b). We next knocked down miR-197 in HEC-1-B cells and overexpressed miR-197 in Ishikawa cells (Figure 5c). A potential binding sequence between hsa_circ_0039569 and miR-197 was predicted by Circular RNA Interactome (Figure 5d). RIP results showed that miR-197 and hsa_circ_0039569 were enriched in Ago2 immunoprecipitates (Figure 5e), indicating their interaction in tumor cells. The binding of hsa_circ_0039569 and miR-197 was also validated by dual luciferase reporter assay. As shown in figure 5f, when the wild-type binding sequence of hsa_circ_0039569 was cotransfected with the miR-197 inhibitor, the luciferase activity was increased. In contrast, when the wild-type binding sequence of hsa_circ_0039569 was cotransfected with miR-197 mimics, the relative luciferase activity was decreased. However, when the mutated binding sequence of hsa_circ_0039569 was cotransfected with either miR-197 mimics or miR-197 inhibitor, the relative luciferase activity did not show a significant difference compared to the NC group. Furthermore, we demonstrated that overexpression of hsa_circ_0039569 substantially downregulated miR-197 expression, while silencing hsa_circ_0039569 significantly upregulated miR-197 expression (Figure 5g). Together, these findings verified that hsa_circ_0039569 sponged miR-197 and attenuated the expression of miR-197.

### Hsa_circ_0039569 increased the expression of HMGA1 by targeting miR-197

**Figure 6.**
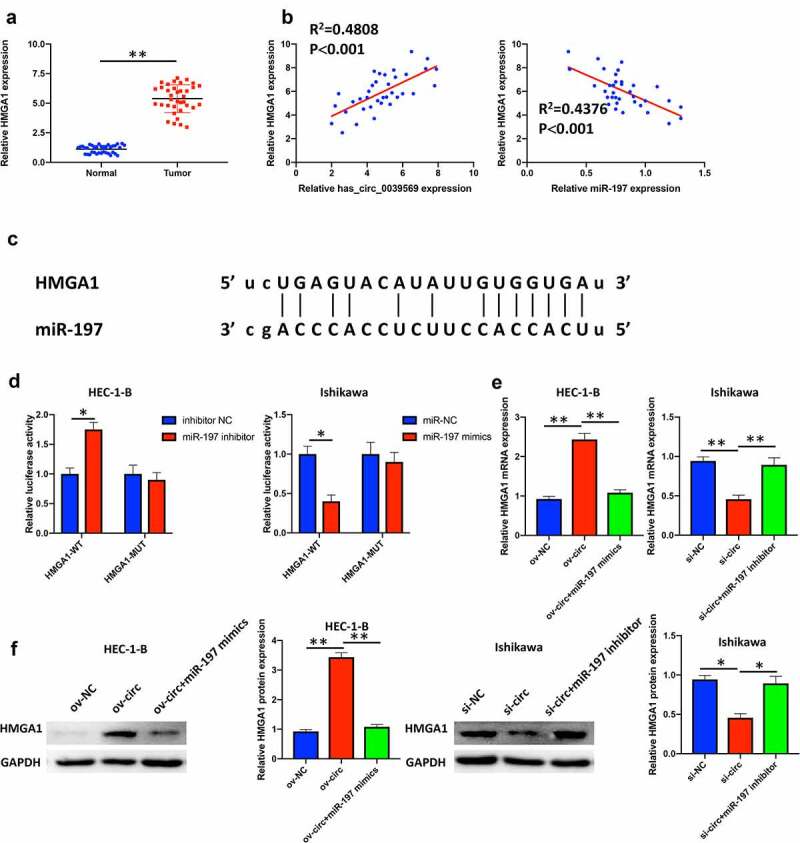
**Hsa_circ_0039569 regulated the expression of HMGA1 by targeting miR-197**. (a) Expression of HMGA1 in normal tissues and endometrial carcinoma tissues was detected by qRT–PCR. (b) Correlations between miR-197 and HMGA1 or HMGA1 and hsa_circ_0039569 were assessed by Pearson’s correlation coefficient test. (c) Illustration of the predictive binding site between miR-197 and HMGA1. (d) A dual luciferase reporter assay was performed to verify the interaction of miR-197 and HMGA1. (e and f) Hsa_circ_0039569 was overexpressed in HEC-1-B cells with miR-197 mimics or was knocked down in Ishikawa cells with a miR-197 inhibitor. The level of HMGA1 was evaluated by qRT–PCR (e) and Western blot (f). * denoted *P* < 0.05, ** denoted *P* < 0.01.

HMGA1 was confirmed as a potential prognostic factor in endometrial carcinoma [20]. In our study, we revealed that HMGA1 was notably elevated in endometrial carcinoma tissues (Figure 6a). Moreover, correlation analysis suggested that HMGA1 was positively associated with hsa_circ_0039569 and negatively associated with miR-197 (Figure 6b). The potential binding between HMGA1 and miR-197 was predicted by starBase (Figure 6c). A dual luciferase reporter assay showed that when the wild-type binding sequence of HMGA1 was cotransfected with the miR-197 inhibitor, the luciferase activity was upregulated. In contrast, when the wild-type binding sequence of HMGA1 was cotransfected with miR-197 mimics, the relative luciferase activity was dramatically downregulated. However, when the mutated binding sequence of HMGA1 was cotransfected with either miR-197 mimics or miR-197 inhibitor, the relative luciferase activity did not show a significant difference compared to the respective NC group (Figure 6d). To verify whether hsa_circ_0039569 regulated the expression of HMGA1 by sponging miR-197, we simultaneously overexpressed hsa_circ_0039569 and miR-197 in HEC-1-B cells or simultaneously knocked down hsa_circ_0039569 and miR-197 in Ishikawa cells. The results revealed that overexpression of hsa_circ_0039569 significantly increased the expression of HMGA1, while overexpression of miR-197 reversed this change. Consistently, silencing hsa_circ_0039569 substantially downregulated HMGA1 expression, while inhibition of miR-197 partially reversed this change (Figures 6e and 6f). Collectively, these data demonstrated that hsa_circ_0039569 upregulated the expression of HMGA1 by targeting miR-197.

*Hsa_circ_0039569 regulated the proliferation of endometrial carcinoma cells via the miR-197/HMGA1 axis*

**Figure 7.**
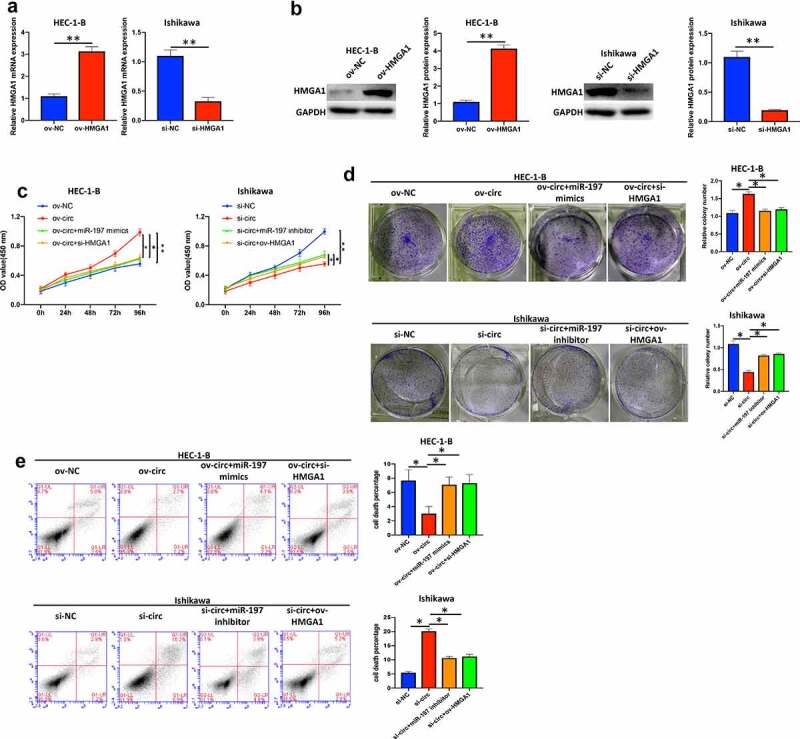
**Hsa_circ_0039569 regulated cell proliferation in endometrial carcinoma via the miR-197/HMGA1 axis**. (a and b) HMGA1 was knocked down by siRNA in HEC-1-B cells or overexpressed in Ishikawa cells. The level of HMGA1 was evaluated by qRT–PCR (a) and Western blot (b). (C to E) Hsa_circ_0039569 was overexpressed in HEC-1-B cells with miR-197 mimics or HMGA1 knockdown. Hsa_circ_0039569 was knocked down in Ishikawa cells overexpressing miR-197 inhibitor or HMGA1. (c) Cell viability was determined by CCK8. (d) A colony formation assay was performed to determine cell proliferation. (e) Cell apoptosis was evaluated by flow cytometry. * denoted *P* < 0.05, ** denoted *P* < 0.01.

First, we overexpressed HMGA1 in HEC-1-B cells and knocked down HMGA1 in Ishikawa cells (Figures 7a and 7b). As shown in Figures 7c and 7d, overexpression of miR-197 or silencing HMGA1 partly reversed the promotive effect of hsa_circ_0039569 overexpression on HEC-1-B cell proliferation, whereas inhibition of miR-197 or HMGA1 overexpression compromised the inhibitory effect of hsa_circ_0039569 knockdown on Ishikawa cell proliferation, as indicated by CCK-8 and colony formation assays. In addition, we also validated that miR-197 mimics or si-HMGA1 compromised the effect of ov-circ on HEC-1-B cell apoptosis, whereas miR-197 inhibitor or ov-HMGA1 compromised the promotive effect of si-circ on Ishikawa cell apoptosis (Figure 7e). Taken together, these results suggested that the miR-197/HMGA1 axis was involved in the regulatory work of hsa_circ_0039569 in cell proliferation.

*Hsa_circ_0039569 modulated the migration and invasion of endometrial carcinoma cells via the miR-197/HMGA1 axis*

**Figure 8.**
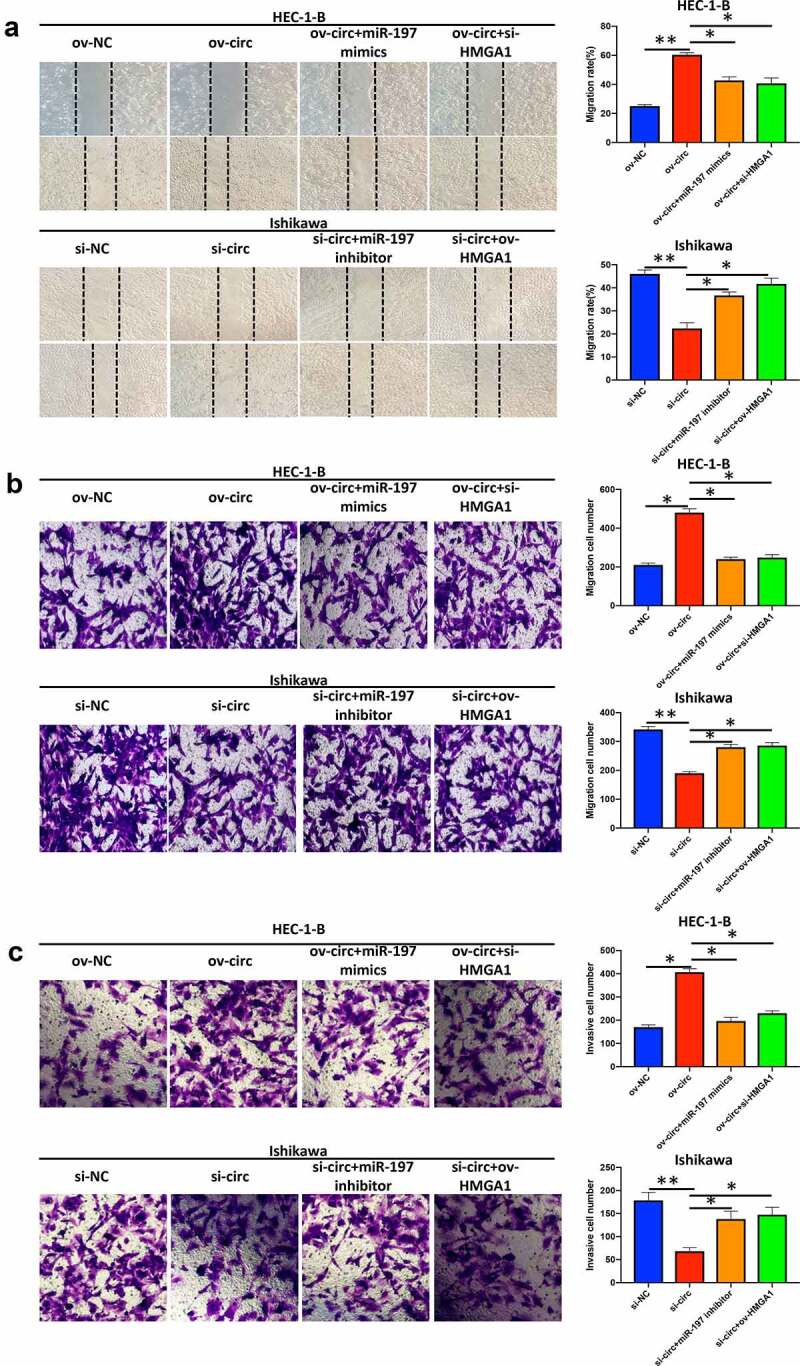
**Hsa_circ_0039569 regulated the migration and invasion of endometrial carcinoma cells via the miR-197/HMGA1 axis**. Hsa_circ_0039569 was overexpressed in HEC-1-B cells with miR-197 mimics or HMGA1 knockdown. Hsa_circ_0039569 was knocked down in Ishikawa cells overexpressing miR-197 inhibitor or HMGA1. (a and b) Cell migrative ability was assessed by wound healing (a) and Transwell (b) assays. (c) Cell invasive ability was assessed by Transwell assay. * denoted *P* < 0.05, ** denoted *P* < 0.01.

Finally, we detected whether hsa_circ_0039569 regulated migration and invasion via the miR-197/HMGA1 axis. The results showed that overexpression of hsa_circ_0039569 promoted the migration of HEC-1-B cells, while simultaneous overexpression of miR-197 or HMGA1 knockdown partially reversed the effect of ov-circ. Accordingly, knockdown of hsa_circ_0039569 in Ishikawa cells weakened the migrative ability of cells, while simultaneous knockdown of miR-197 or HMGA1 overexpression restored the migrative ability (Figures 8a and 8b). Similarly, we found that overexpression of hsa_circ_0039569 promoted the invasion of HEC-1-B cells, while simultaneous overexpression of miR-197 or HMGA1 knockdown compromised the effect of hsa_circ_0039569 overexpression. Meanwhile, hsa_circ_0039569 knockdown in Ishikawa cells impaired their invasive ability, while knockdown of miR-197 or HMGA1 overexpression restored the invasive ability (Figure 8c). The above findings indicated that hsa_circ_0039569 promoted cell migration and invasion in endometrial carcinoma via the miR-197/HMGA1 axis.

## Discussion

Endometrial carcinoma is a group of epithelial malignancies occurring in the endometrium, most commonly in perimenopausal and postmenopausal women [21]. Endometrial carcinoma is one of the most common cancers of the female reproductive system, accounting for nearly 200,000 new cases each year, and endometrial carcinoma is the third most common cause of death from gynecological malignancies [3]. Targeted therapies that suppress cell proliferation and metastasis are considered to be a breakthrough in the treatment of human cancers. Due to the clinical and functional relevance of circRNAs in tumor progression, research on circRNAs is receiving extensive attention. In the current study, we identified a novel circular RNA, hsa_circ_0039569, that was upregulated in endometrial carcinoma. Hsa_circ_0039569 facilitated cell proliferation, migration and invasion of endometrial carcinoma cells *in vitro*. Moreover, hsa_circ_0039569 promoted endometrial carcinoma progression by sponging miR-197 and thus upregulating the expression of HMGA1.

Recently, circRNAs were revealed as biomarkers and regulators in endometrial carcinoma. CircTNFRSF21 [6], circ_0000043 [8], hsa_circ_0061140 [7], hsa_circ_0002577 [14], and circ_PUM1 [22] were all elevated in endometrial carcinoma and facilitate the development of endometrial carcinoma by targeting different functional axes. Here, we demonstrated that hsa_circ_0039569 was also elevated in endometrial carcinoma tissues and cell lines. Similarly, hsa_circ_0039569 was also shown to be an oncogenic regulator in endometrial carcinoma. These findings are supported in the study by Ye et al., in which hsa_circ_0039569 was upregulated in endometrial carcinoma [10] and are consistent with a previous study in renal cell carcinoma, which indicated that hsa_circ_0039569 led to an increase in proliferation and metastasis by targeting the miR-34a-5p/C-C motif chemokine ligand 22 (CCL22) axis [23]. However, the functional role of hsa_circ_0039569 in other cancer types remains largely unknown and needs further investigation.

CircRNAs can serve as competitive endogenous RNAs (ceRNAs) to sponge miRNAs and impair the suppressive function of miRNAs on downstream mRNAs [24]. In our study, we illustrated that hsa_circ_0039569 sponged miR-197 to upregulate HMGA1. Rescue experiments verified that the miR-197/HMGA1 axis was involved in the regulatory work of hsa_circ_0039569 in endometrial carcinoma. However, we should be cautious as hsa_circ_0039569 might exhibit oncogenic function by sponging numerous miRNAs, which should be investigated in our further work. In addition to sponging miRNAs, circRNAs can regulate the expression of downstream genes via other mechanisms. For example, circ-CCAC1 can sequester the enhancer of zeste 2 polycomb repressive complex 2 subunit (EZH2) in the cytoplasm to elevate the expression of SH3GL2 [25]. Circular RNA circ-ADD3 can facilitate degradation of EZH2 via ubiquitination [26]. These studies remind us that hsa_circ_0039569 might regulate the expression of genes through a miRNA-independent mechanism, such as binding with EZH2 or RNA binding proteins.

A previous study suggested that lower miR-197 was found in endometrial carcinoma and that miR-197 mediated the functional role of hsa_circ_0002577 [14]. In this work, we consistently validated the decrease in miR-197 in endometrial carcinoma. Moreover, we revealed that miR-197 was sponged by a novel circRNA, hsa_circ_0039569, and targeted HMGA1 to reduce the expression of HMGA1 mRNA. Overexpression of HMGA1 might be a potential prognostic factor in endometrial carcinoma [20]. HMGA1 can promote tumor progression of endometrial carcinoma by activating Wnt/β-catenin signaling [27]. Additionally, HMGA1 regulates angiogenesis in breast cancer and chemoresistance in gastric cancer [28,29]. These findings suggested that the hsa_circ_0039569/miR-197/HMGA1 axis might play a role in other malignant phenotypes of endometrial carcinoma, which needs further investigation. Hsa_circ_0039569 might facilitate angiogenesis and chemoresistance in endometrial carcinoma.

## Limitations

The major limitation of our present work is that the findings were based on *in vitro* studies. No *in vivo* animal study was performed to support the conclusion. In our further work, we should use *in vivo* tumorigenesis and metastasis models to validate our *in vitro* findings.

## Conclusion

Taken together, these results demonstrated that hsa_circ_0039569 may play an oncogenic role in endometrial carcinoma progression by acting as a sponge of miR-197 and upregulating HMGA1 expression, which might support hsa_circ_0039569 as a potential therapeutic target for endometrial carcinoma.

## Data Availability

The datasets used or analyzed in the current study are available from the corresponding author on reasonable request.

## Highlights

1. hsa_circ_0039569 was highly expressed in endometrial carcinoma

2. hsa_circ_0039569 sponged miR-197 to upregulate HMGA1 expression

3. hsa_circ_0039569 regulated cell proliferation, migration and invasion in endometrial carcinoma by targeting the miR-197/HMGA1 axis
